# Solution Synthesis, Processing, and Applications of Semiconducting Nanomaterials

**DOI:** 10.3390/nano9101442

**Published:** 2019-10-11

**Authors:** Julia W. P. Hsu

**Affiliations:** Department of Materials Science and Engineering, University of Texas at Dallas, Richardson, TX 75080, USA; jwhsu@utdallas.edu

Nanomaterials have contributed to the forefront of materials research in the past two decades, and are used today in sensors, solar cells, light emitting diodes, electronics, and biomedical devices. Solution synthesis and processing offer inexpensive, low-temperature, energy efficient, and environmentally friendly approaches that are desired especially for mass production or integration with plastic substrates. While metal nanoparticles, in particular gold, have been researched widely, semiconducting nanomaterials offer much greater versatility, functionality, and applications. The frontiers in synthesis include new compounds, reducing the size of nanomaterials, introducing new morphologies from assembly or templating, alternative green synthesis methods to reduce waste and energy, and surface functionalization. Furthermore, great challenges are encountered in processing nanomaterials from suspensions to thin films on different substrates for device applications. While many publications focus on synthesis and applications of solution-based nanomaterials, issues related to processing, e.g., solvent choice, surface compositions and ligands, particle–particle interaction, and deposition methods, are infrequently addressed. This Special Issue includes nine articles and one review, covering synthesis [1], novel processing [2,3,4,5], and a wide range of applications, such as solar cells [1,3,6], sensors [7], catalysis [8], and electronics [9,10].

Cu_2_ZnSnS_4_ (CZTS) is a kesterite material for solar cells based on earth abundant elements. An ecofriendly method to fabricate CZTS films is much sought after. Zhang et al. [1] adopted a wet ball milling method, which uses only nontoxic solvents. The films made from as-fabricated CZTS nanocrystal inks were followed by a rapid high-pressure sulfur annealing step to promote grain growth and crystallinity. They achieved solar cell efficiency of 6.2% (open circuit voltage: *V*oc = 633.3 mV, short circuit current: *J*sc = 17.6 mA/cm^2^, and fill factor: FF = 55.8%) with an area of 0.2 cm^2^. Replacing sulfur with selenium in a kesterite material lowers its bandgap to better match the solar spectrum. Jiang et al. [3] investigated selenization treatment on Cu_2_Mg_0.2_Zn_0.8_Sn(S,Se)_4_ (CMZTSSe) films made by a sol–gel method. By controlling the selenization temperature and time, the crystallinity, film morphology, band gap, and hole concentration can be tuned. An optimized processing condition was reported.

Delafossite materials, with a chemical formula of AMO_2_ (A = Cu or Ag and M is a trivalent ion), are rare p-type oxides. It was first reported in 1997 that CuAlO_2_ was a true p-type transparent conducting oxide, which stimulated many activities in delafossite materials [11]. This Special Issue includes two articles on CuCrO_2_ (CCO). Wu et al. [5] made novel Al_2_O_3_-CCO core-shell nanofibers and CCO hollow nanotubes. The CCO was grown on the surface of electrospun Al_2_O_3_ fibers by a solution method followed by annealing at 600 °C in a vacuum, resulting in Al_2_O_3_-CCO core-shell nanofibers. By applying sulfuric acid to the core-shell nanofibers, the authors showed that the Al_2_O_3_ cores were selectively removed while the rest of the original structures were preserved, hence producing CCO hollow nanotubes with an inner diameter of 70 nm and a wall thickness of 30 nm. Zhang et al. [6] introduced Mg as a dopant in CCO nanoparticles and examined the effects on the performance of solar cells using these materials as the hole transport layer (HTL). CCO nanoparticles have been shown as an efficient HTL in dye sensitized solar cells [12], organic solar cells [13], and, recently, halide perovskite solar cells [14,15,16,17]. This work was the first demonstration of how CCO and Mg-doped CCO perform as efficient HTLs for a non-fullerene acceptor bulk heterojunction (BHJ) system. They also observed Mg doping results in a small but definitive increase in the short circuit current density for all active layer systems, including three different BHJs and halide perovskite.

In addition to CCO hollow nanotubes, complex nanostructures have many interesting properties and applications. Bhalothia et al. [8] reported on the synthesis and oxygen reduction reaction activity of Au-cluster-decorated NiO_x_@Pt nanostructures. They found an impressive performance of these nanocatalysts compared to commercial benchmarks: A 17-times larger kinetic current and a 53-fold increase in specific activity. Wu et al. [7] fabricated ammonia sensors based on self-assembly SnO nanoshells via a solution method that can detect ammonia below 20 ppm with high selectivity. Last but not least, Yun and Paik [4] contributed a review to the Special Issue on self-assembly of inorganic nanocrystals into superlattice thin films and multiscale nanostructures. The paper includes diverse examples of highly ordered superlattices.

Intense pulsed light (IPL) irradiation shows promise in rapid material processing that is compatible with roll-to-roll manufacturing [18]. Nakamura et al. [2] synthesized Cu nitride nanoparticles as an ink and converted them to Cu wires using IPL processing. This approach allows the production of large quantities of printed circuit boards with less waste. IPL also has the potential for device fabrication on low-temperature plastic substrates. Other electronic applications in this Special Issue includes flexible HfO_2_ memory devices and indium-gallium-zinc oxide (IGZO) thin film transistors (TFTs). Liu et al. [9] prepared a novel flexible Au/HfO_2_/Pt resistive random access memory devices on a mica substrate using a sol–gel process. Moreira et al. [10] investigated solution processed IGZO films to replace vacuum deposition to implement low-cost, high-performance electronic devices on flexible transparent substrates. They evaluated the influence of composition, thickness, and aging on the electrical properties of IGZO TFTs, using a solution combustion synthesis method with urea as fuel. The optimized TFT built on a solution-processed AlO_x_ dielectric showed a saturation mobility of 3.2 cm^2^ V^−1^ s^−1^, an on–off ratio of 10^6^, a sub-threshold swing of 73 mV dec^−1^, and a threshold voltage of 0.18 V, thus demonstrating promising features for low-cost circuit applications.

I hope *Nanomaterials* readers find these articles informative and interesting.
